# Advances in functional genomics for investigating salinity stress tolerance mechanisms in cereals

**DOI:** 10.3389/fpls.2013.00123

**Published:** 2013-05-10

**Authors:** Megan C. Shelden, Ute Roessner

**Affiliations:** Australian Centre for Plant Functional Genomics, School of Botany, University of MelbourneParkville VIC, Australia

**Keywords:** abiotic stress tolerance, functional genomics, spatial resolution, metabolomics, expression profiling

## Abstract

Abiotic stresses such as low water availability and high salinity are major causes of cereal crop yield losses and significantly impact on sustainability. Wheat and barley are two of the most important cereal crops (after maize and rice) and are grown in increasingly hostile environments with soil salinity and drought both expected to increase this century, reducing the availability of arable land. Barley and wheat are classified as glycophytes (salt-sensitive), yet they are more salt-tolerant than other cereal crops such as rice and so are good models for studying salt tolerance in cereals. The exploitation of genetic variation of phenotypic traits through plant breeding could significantly improve growth of cereals in salinity-affected regions, thus leading to improved crop yields. Genetic variation in phenotypic traits for abiotic stress tolerance have been identified in land races and wild germplasm but the molecular basis of these differences is often difficult to determine due to the complex genetic nature of these species. High-throughput functional genomics technologies, such as transcriptomics, metabolomics, proteomics, and ionomics are powerful tools for investigating the molecular responses of plants to abiotic stress. The advancement of these technologies has allowed for the identification and quantification of transcript/metabolites in specific cell types and/or tissues. Using these new technologies on plants will provide a powerful tool to uncovering genetic traits in more complex species such as wheat and barley and provide novel insights into the molecular mechanisms of salinity stress tolerance.

## INTRODUCTION

Abiotic stresses such as drought, salinity, extreme temperatures and nutrient deficiency, and toxicity are major causes of plant stress and agriculture yield losses. With an increase in the loss of arable land due to drought and salinity it is imperative that we increase the crop yield per hectare. In terms of worldwide production maize, rice, wheat, and barley are the four most important crops (844, 672, 650, and 123 MT, respectively, 2010^1^. Wheat and barley are grown in increasingly hostile environments with soil salinity and drought both expected to increase this century reducing the availability of arable land. With the global population expected to reach nine billion by 2050^2^ an increase in global agriculture productivity will be needed to meet the increase in demand for global food supply. Around 80% of human food produced is comprised of crops, with this dominated by the cereals that comprise 50% of global food production (Langridge and Fleury, 2011). Since the 1960s global cereal production has undergone a linear increase of 30% per year (Tester and Langridge, 2010).However, in order to meet the required increase in food production, crop productivity will need to increase by 38% annually. To achieve this increase in food production it will be imperative to look for genetic improvement of crops, not only by using traditional plant breeding methods but also genetic modification technologies.

Complex traits such as an increased tolerance to abiotic stresses are multi-genic and thus are difficult to identify. The improvement of crops is largely dependent on exploiting genetic variation and this has been achieved in the past through traditional plant breeding methods (Langridge and Fleury, 2011). One approach to elucidate abiotic stress tolerance mechanisms is to identify naturally occurring genetic variation within a crop species by screening varieties, wild genotypes and landraces (Roy et al., 2011b). The use of forward genetics is a powerful approach to study traits contributing to abiotic stress tolerance, however, it is crucial that reliable and accurate quantitative phenotyping methods are employed to assist in the identification of these traits. In recent years, phenomics technologies have become much more advanced and high-throughput, non-destructive measurements can now be made to assist in accurate phenotyping (reviewed in Roy et al., 2011b). In conjunction, recent advances in high-throughput functional genomics technologies such as next generation sequencing (NGS), transcriptomics, metabolomics, and proteomics make it possible to use a systems biology approach to understand the response to environmental stress. In this review we will discuss the use of high-throughput functional genomics technologies to understand salinity stress tolerance mechanisms in plants focussing on cereal crops. Most functional genomics studies have used the model plant *Arabidopsis* and very few studies have been conducted on more genetically complex plant species such as wheat. However, with the rapid development of functional genomic tools we can now use this technology to identify abiotic stress tolerance mechanisms in cereal crops. We also highlight the need to use both spatial and temporal resolution to elucidate the molecular response to salinity (and other abiotic stresses). These studies will lead to a greater understanding of the plant response to salinity stress and thus the integration of this data will contribute to improving our ability to generate salt-tolerant crops.

## GENETIC VARIATION INSALT STRESS TOLERANCE IN CEREAL CROPS

Different plant species have a wide ranging capacity for salt tolerance from the very sensitive species (glycophytes) such as the model plant *Arabidopsis* to the very tolerant halophyticspecies such as *Atriplex * spp. (saltbush). Cereal crops are classified as glycophytes, however, different crop species can also have different capacities and mechanisms to tolerate salt stress, for example, rice is more sensitive than both barley and wheat(Munns and Tester, 2008). Within a species there can also be naturally occuring genetic variation in salt tolerance and this can be exploited for breeding of salt-tolerant crops (Roy et al., 2011b). It has been proposed that the temporal response of plantsto soil salinity occurs in two separate phases that have been termed as osmotic and ionic (Munns, 2002).The early phase (hours to days) is described as an osmotic stress, due to the low water potentialaround the roots, in response to saline soils and/or water deficit. Osmotic stress is described as a shoot ion independent stress and can result in cell dehydration and loss of cell turgor pressure, and can be characterized phenotypically by a reduction in root elongation, inhibition of photosynthesis and a reduction in shoot growth (Munns and Tester, 2008).In contrast, ionic stress occurs at a later stage (usually after weeks or months) and is a result of the accumulation of toxic concentrations of Na^+^ and Cl^-^ in the cell cytoplasm resulting in decreased growth and yield. In response to salt stress, crop plants have evolved the following three tolerance mechanisms – (1) osmotic stress tolerance: ability to maintain water uptake and growth, (2) Na^+^ exclusion: exclusion of toxic ions from the shoot tissues, and (3) tissue tolerance: compartmentalisation of toxic ions into the vacuole or specific tissues (Munns and Tester, 2008).

**FIGURE 1:**
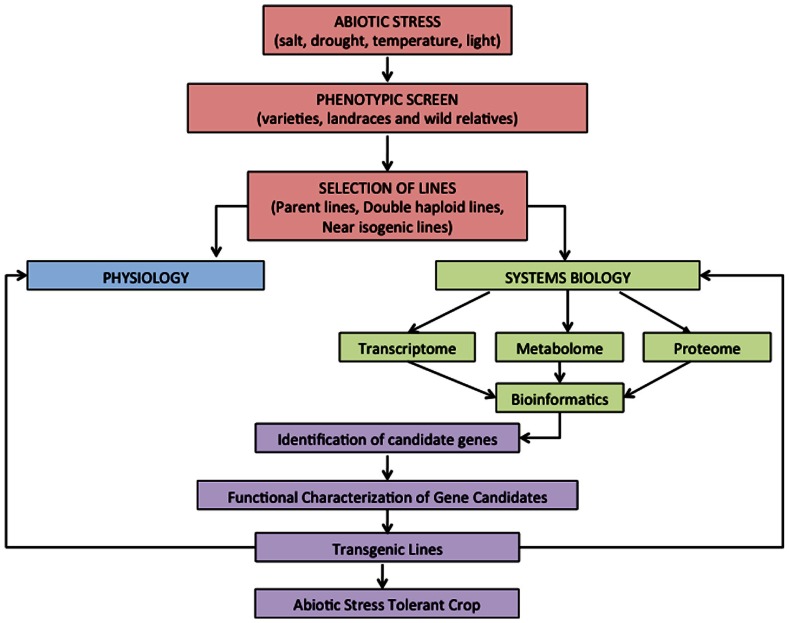
**Proposed strategy for the integration of physiology and systems biology to gain insights into abiotic stress responses in cereals and the future development of abiotic stress tolerant crops**.

One approach to identify adaptive traits to abiotic stresses is to screen for genetic diversity in populations (**Figure 1**). Genetic variation in salt tolerance has been shown inmany cereal crops including barley (Shavrukov et al., 2010) and wheat (James et al., 2008; Rahnama et al., 2011).The focus of most research for enhancing salt tolerance in plants has concentrated on the mechanisms that control Na^+^ exclusion (Munns and Tester, 2008). For example, in a study on durum wheat genotypes, a wide genetic variation was observed in their ability to exclude Na^+^ that was not present in modern cultivars (Munns et al., 2000). This led to the identification of two major genes for Na^+^ exclusion, *Nax1* and *Nax2 * (Lindsay et al., 2004; James et al., 2006). The intergression of *Nax2* from the parent line, *Triticum monococcum* , into durum wheat produced a salt-tolerant phenotype (James et al., 2012).

It was originally thought that there was very little evidence of genetic variation in osmotic stress tolerance (Munns et al., 1995), so this area of research has largely been neglectedand there are limited studies reporting genetic variation in osmotic stress tolerance within a cereal species. A number of recent studies have identified genetic variation in osmotic stress tolerance by measuring shoot traits. In a study of 12 *Triticum monococcum * accessions, non-destructive assays were developed to distinguish the three mechanims of salinity tolerance; osmotic tolerance, Na^+^ exclusion and tissue tolerance (Rajendran et al., 2009). Different accessions appear to use different combinations of the three tolerance mechanisms to increase their salinity tolerance. This information can now be used for forward genetic screens to identify the molecular basis of these tolerance mechanims.

Stomatal conductance is known to be reduced immediately upon exposure of the roots to salinity, so the plant must be responding systemically to the osmotic impact.For example, in a screen of 50 durum varieties and landraces, two- to threefold differences in stomatal conductance were observed and higher stomatal conductance positively correlated with relative growth rate (James et al., 2008). Despite a number of recent phenotypic studies, the molecular mechanisms for osmotic stress tolerance remain unknown. Osmotic stress is initially detected by the roots upon exposure to a low water potential (either as a result of water deficit or salinity). There are few studies decribing genetic variation in the root growth response to osmotic stress, mainly due to the difficulties associated with visualizing and phenotyping roots (Gewin, 2010; Richards et al., 2010; Zhu et al., 2011). One example of genetic variation in root growth has been shown by the measurement ofroot elongation rate of eight barley genotypes (including barley cultivars, a landrace and wild barley) in response to the early component of salinity stress. In this study the wild barley, *Hordeum vulgare ssp. spontaneum* , was identified as the most tolerant (Shelden et al., 2013). The identification of genetic variation in root phenotypes will provide important information for future genetic characterisation and molecular studies to elucidate the molecular pathways involved in the early phase of salinity tolerance.

## UTILIZING FUNCTIONAL GENOMICS APPROACHES TO ELUCIDATE PLANT STRESS RESPONSES

Functional genomics approaches such as genomics, transcriptomics, metabolomics, proteomics, and ionomics, have been extensively used to evaluate abiotic stress tolerance mechanisms in plants (Cramer et al., 2011). These platforms can be utilized to improve our ability to discover the genes and pathways that control specific traits in response to abiotic stress (**Figure 1**).

Plants are complex organisms with many different tissue and cell types and the interaction between these tissues and cells requires complex regulation. To gain a greater understanding of the complexities of cell-specific regulation, an increase in the sensitivity of functional genomic tools are required. To date, functional genomics approaches have mainly been utilized for studies of whole organs such as leaves and roots (Widodo et al., 2009), thus it is possible that important changes in both metabolites and gene expression go undetected because they are diluted out by the surrounding tissue. Combining a forward genetics approach with spatially resolved ‘omics approaches may greatly improve our chances of discovering gene candidates leading to the generation of abiotic stress tolerant crops.

## ADVANCEMENTS IN FUNCTIONAL GENOMICS TECHNIQUES FOR SPATIAL RESOLUTION

### TRANSCRIPTOMICS

Microarrays were the first available method for genome wide transcript expression profiling and have been used extensively in plant biology. Microarrays have been extensively used to generate transcriptional profiles in response to abiotic stresses in a range of plant species including, but not limited to, *Arabidopsis* (Kreps et al., 2002), *Vitis vinifera* L. (grapevine; Cramer et al., 2007) and *Hordeum vulgare* L. (barley; Walia et al., 2006). Studies of the transcriptional response to salt stress have been conducted in barley (Ueda et al., 2004, 2006; Walia et al., 2006, 2007; Gruber et al., 2009) and the model cereal *Brachypodium * (Kim et al., 2012) but due to the polyploidy genome, studies of wheat are more limited (Kawaura et al., 2006, 2008; Mott and Wang, 2007; Jamil et al., 2011; Garg et al., 2013).As the technology of microarrays and cell separation techniques has become more advanced it has become possible to do spatial profiling of plant tissues and cell-specific studies (Brady et al., 2007; Dinneny et al., 2008; Spollen et al., 2008). Cell-type specific transcript studies have been conducted in many plant species including *Arabidopsis* and the cereals maize, rice, barley, and soybean using microarrays (Pu and Brady, 2010; Long, 2011; Rogers et al., 2012). Despite a large number of microarray experiments, very few have led to the identification of stress tolerance pathways (Deyholos, 2010).

High-throughput, deep sequencing technology (RNA-Seq) provides a new strategy to detect and accurately quantitate changes in the transcriptome. NGS is still in its infancy in plant biology but is expected to supercede microarray based approaches as the technology becomes more developed. NGS technology is a powerful tool for gene expression profiling and has been used successfully in a number of organisms including mammalian systems (Mortazavi et al., 2008), yeast (Nagalakshmi et al., 2008) and plants (Lister et al., 2008) to show transcriptional activity. The use of NGS technology for expression studies is becoming more popular with studies reported in the model plant *Arabidopsis* (Weber et al., 2007) and several cereal species including *Glycine max* L. (soybean; Fan et al., 2013), *Lolium perenne* L. (perennial ryegrass; Studer et al., 2012), *Triticum aestivum * (wheat) endosperm (Gillies et al., 2012), *Zea mays * (maize; Li et al., 2010), *Sorghum bicolor * (sorghum; Dugas et al., 2011), *Panicum virgatum* L. (switchgrass; Wang et al., 2012) and the extremophile *Thellungiella parvula * (Dassanayake et al., 2011). Studies now show that RNA-Seq provides better quantitation and accuracy than microarrays (Jain, 2012). Theuse of RNA-Seq in plant biology has been comprehensively reviewed and will therefore not be reviewed here (Jain, 2011, 2012). There are only very few studies published utilizing NGS to study abiotic stress responses in plants (Deyholos, 2010). These include studies on salt stress (Molina et al., 2008; Fan et al., 2013), cold stress (Tamura and Yonemaru, 2010) and drought stress (Dugas et al., 2011; Dong et al., 2012; Vidal et al., 2012).

With the availability of a number of cereal genomes including Barley Sequencing Consortium (2012), sorghum (Paterson et al., 2009), maize (Schnable et al., 2009), rice (Project, 2005), and the cereal model *Brachypodium * (Vogel et al., 2010) it is now possible to use this information to conduct large scale NGS transcriptomic experiments in cereal crops. For example, in a study on *Sorghum bicolor* , RNA-Seq technology was utilized in combination with the sorghum genome sequence to analyze the root and shoot transcriptome in response to osmotic stress and abscisic acid (ABA; Dugas et al., 2011). The study identified over 28,000 unique genes that were transcriptionally regulated in response to osmotic stress and ABA. A collection of drought-regulated gene networks and transcription factors were identified that can provide a basis for further studies. The data in this study was also compared to other sequenced transcriptomes including maize, rice and *Arabidopsis* , identifying more than 50 differentially expressed drought responsive gene orthologs. The lack of fully sequenced genomes in plants with larger, more complex genomes, such as wheat (hexaploid), present many difficulties for these types of ‘omics studies. Utilizing these new technologies in combination with the completed genome sequences of cereals will providea powerful tool to uncovering genetic traits in more complex species such as wheat.

### PROTEOMICS

Proteomic studies of the response to salt stress have been conducted in many plant species including the cereals rice (*Oryza sativa* ), wheat (*Triticum durum* and *Triticum aestivum* ), barley (*Hordeum vulgare* ), and maize (*Zea mays* ; reviewed in Zhang et al., 2012). A number of comprehensive reviews describing proteomic responses to abiotic stress are available and therefore will not be reviewed here (Kosova et al., 2011; Roy et al., 2011a; Zhang et al., 2012). Single cell proteomic studies have been successful in mammalian systems where cells can be cultured to increase the starting material (Schirle et al., 2003; Diks and Peppelenbosch, 2004), but there are relatively few examples of single cell proteomic studies in plants, and especially in cereals, due to the difficulties in obtaining enough material. Single cell-type proteomic analyses have been reported for *Arabidopsis* guard cells (Zhao et al., 2008), trichomes (Wienkoop et al., 2004; Kryvych et al., 2011) and pollen (Holmes-Davis et al., 2005), soybean (*Glycine max* ) root hairs (Wan et al., 2005) and tobacco (*Nicotiana tabacum* ) trichomes (Amme et al., 2005). Tissue specific proteomic studies of the response to salinity include a comprehensive proteomics study of rice anthers (Sarhadi et al., 2012) wheat root seedlings (Guo et al., 2012) and rice plasma membranes (Cheng et al., 2009). Kamal et al. (2012) analyzed wheat chloroplast protein abundance only and correlated their salt-responsive behavior to numerous physiological parameters. However, in most of these studies only a small number of salt-responsive proteins were identified (Zhao et al., 2008).

### METABOLOMICS

Metabolites are the building blocks for structural and enzymatic molecules and carry the energy required for growth and maintenance of living cells. Metabolites are a key link between genetic information and a phenotype and are a measure of the physiological state of an organism. In the past decade enormous efforts have been made to develop high-throughput and untargeted methodologies to analyze as many metabolites as possible. The resulting relatively new field of metabolomics uses a set of sophisticated analytical biochemistry tools for the identification and quantification of metabolites. The application of bioinformatics and statistical tools allows the extraction and analysis of multivariate data sets supporting biological interpretation. An inherent challenge of metabolomics derives from the fact that the metabolome is a simple descriptor for what is, in fact, huge chemical diversity. Hundreds and hundreds of compounds with different physical and chemical properties such as molecular weight, molecular size, polarity, stability, volatility, solubility, and many more, require the development of comprehensive and/or complementary extraction, separation, detection, and quantification techniques (Beckles and Roessner, 2012). A number of analytical technologies, including chromatographic separation techniques, such as liquid and gas chromatography, coupled to mass spectrometry and nuclear magnetic resonance (NMR) spectroscopy have been successfully used to analyze metabolites in many different organisms, tissues and biofluids (Roessner and Beckles, 2009).

Metabolomic analysis has been used extensively to study abiotic stress responses in plants (Urano et al., 2010; Obata and Fernie, 2012). Due to the importance of salinity and drought stress in agriculture, many metabolomic studies have been conducted on agriculturally important crops to gain insight into the effect of such stress on metabolic activity. Analyzing genotypes that have differing phenotypic responses to abiotic stress (sensitive vs. tolerant) will help elucidate the specific metabolic changes that are contributing to the increased tolerance of a genotype. For example, in a study conducted on two genotypes of barley that differ in salt tolerance, it was found that the landrace Sahara accumulates higher levels of metabolites involved in cellular protection in the leaves, corresponding with higher leaf Na^+^ concentrations compared to the less tolerant genotype, Clipper (Widodo et al., 2009).

To date, most metabolomics analyses have been carried out on bulked tissues (e.g., whole roots or leaves) due to sensitivity levels of the technologies employed. This means that the metabolic profile of different tissue and cell types, each likely to be characterized by a specific metabolite profile or a particular response to environmental or genetic stimuli, have to be measured together. Instrumentation with increased sensitivity will help substantially, but a major problem remains, that it is often difficult or even impossible to separate and isolate tissue types or single cell types from plant tissues. The first success of a single cell metabolomics approach has been reported in a study of *Arabidopsis * where cryo-sectioning was used to preserve cellular structures. Specific cell types were cut and collected using laser micro-dissection to obtain a sufficient number of cells to allow the detection of about 68 major metabolites by gas chromotography/mass spectrometry (GC-MS; Schad et al., 2005). In future, it may be possible to develop cell-type specific protoplasts that can be cultured to increase the amount of tissue for analysis (Keerberg et al., 2011). However, one issue in any such metabolomics analysis is that protoplasts are cell-wall free cells and their generation and culture may affect the physiological state of the cells and therefore the range and concentration of metabolites.

An important aim of the characterization of metabolites in plants is to understand the spatial distribution of single metabolites. Initial such analyses have includedindole-3-acetic acid (IAA) in *Arabidopsis* roots (Petersson et al., 2009) or ATP in *Vicia faba* embryos (Borisjuk et al., 2003), but these studies are now being extended to as many metabolites as possible. A very promising new approach to the spatial analysis of both proteins and metabolites is to raster across thin tissue slices and ionize any compound (peptides following proteolytic digestion or metabolites) using matrix-assisted laser desorption ionization (MALDI) coupled with analysis of every ionized molecule in a mass spectrometer (Kaspar et al., 2011). This technique, although not having cellular resolution, allows the determination of tissue-type metabolite distributions. This approach has been well developed for lipids (Vrkoslav et al., 2010; Horn et al., 2012) since they are easily ionized, while efforts are now being made to optimize this approach for proteins/peptides (Grassl et al., 2011) and small molecules (Jun et al., 2010; Lee et al., 2012; Peukert et al., 2012). However, there are currently no reports in the literature on applications of metabolomics to the spatial analysis of salinity responses in plants. Therefore this approach provides the potential to monitor the cell and tissue specific adaptation mechanisms in cereals and provide novel ideas for the development of more tolerant crop genotypes. Techniques such as laser micro-dissection (Yi et al., 2012), fluorescence-activated cell sorting (FACS; (Borges et al., 2012; Evrard et al., 2012) or MALDI based mass spectral imaging (Kaspar et al., 2011) may pave the way for such highly spatially resolved analyses.

### IONOMICS

The high-throughput analysis of elemental compositions is called ionomics and has been extensively used in the plant sciences for screening mutant collections, forward and reverse genetics approaches and investigating mechanisms of elemental or ion uptake, transport, compartmentalisation and exclusion (Baxter, 2009). In the area of abiotic stress research, elemental profiling provides a way to investigate a plant’s response to salinity, nutrient deficiencies and toxicities or hyper-accumulation. There are 13 minerals essential to all plants, including macronutrients, such as N and P, and micronutrients such as Na, K, B, Mn, Fe, and Ca. Several mineral deficiencies lead to dramatic growth retardation and possibly death, but excess amounts of some minerals can also be toxic. In both cases, plant metabolism is dramatically affected; plants are able to develop mechanisms in order to cope with either deficiency or toxicity. Analysis of the elemental composition is not only important when studying effects of high salt on a plant but also when identifying mechanisms of adaptation and tolerance to any osmotic stress such as drought. Some ions have hygroscopic properties and therefore are utilized to retain water in the cell in order to sustain turgor pressure upon osmotic stress such as water deficiency. There have been many reports on the effects of the expression of ion transporters in plants; studies aimed at determining the function of those transporters (Horie et al., 2009; Jiang et al., 2010; Xue et al., 2011) or increasing tolerance to either salinity or nutrient deficiencies or toxicities (Hauser and Horie, 2010; Plett and Moller, 2010; Horie et al., 2011; Mian et al., 2011).

The simultaneous analysis of the major macro-elements and many of the microelements can be achieved using inductively coupled plasma spectroscopy or optical emission spectroscopy coupled with mass spectrometry (ICP-MS and OES-MS, respectively). With appropriate sample preparation methods, these techniques allow the analysis of between 12 and 20 elements and ions simultaneously (Baxter, 2009). However, in most studies bulk tissue (e.g., leaves or roots) is extracted for elemental analysis, thus giving no information about spatial distribution of elemental composition. Emerging technologies that allow image distributions of elemental composition of biological tissues have now become available for plant tissue sections. Laser ablation inductively coupled plasma mass spectrometry (LA-ICP-MS) is a very sensitive and efficient technique that produces three-dimensional images of spatial element distributions in thin biological tissue sections (Becker, 2010). This well advanced technique for the analysis of elemental distributions in human or animal tissue sections allows the reconstruction of three-dimensional atlases of spatial concentrations of elements (Hare et al., 2012). In the plant sciences this technique has not been utilized extensively but it is easy to envisage using it in numerous productive applications.

### EXAMPLES OF SPATIALLY RESOLVED ‘OMICS FOR IDENTIFYING ABIOTIC STRESS TOLERANCE MECHANISMS IN CEREALS

There are very few examples of using spatially resolved functional genomics technologies to identify abiotic stress tolerance mechanisms in cereal crops. Utilizing emerging high resolution technologies to profile changes in a defined region of the plant could lead to novel insights into the molecular mechanisms of abiotic stress tolerance. The* Arabidopsis * root has been used as a model for understanding the mechanisms of cell-specific processes and has been extensively reviewed by Dinneny (2010). The development of green fluorescent protein (GFP) reporter lines enabled the characterization of 14 different cell types in the* Arabidopsis * root, revealing a complex pattern of spatial control of biological functions (Brady et al., 2007). The advancement of these technologies and our current understanding from model systems such as *Arabidopsis * can now be utilized to increase our understanding of more complex plant systems such as the cereal crops. This will allow development of crops with enhanced tolerance mechanisms to environmental stresses.

The importance of using spatial resolution for ‘omics studies of abiotic stress has been shown in a study on maize roots in response to water deficit (Yamaguchi and Sharp, 2010). The maize primary root has been extensively studied as a model crop system for understanding the root growth response to water deficit. Physiological studies demonstrated that the response to osmotic stress is not uniform in the apical region of the root; the root elongation rate varies spatially in the elongation zone in response to both water deficit (Sharp et al., 1988) and salinity (Bernstein and Kafkafi, 2002). Physiological studies were combined with transcriptomic and proteomic profiling for spatial and temporal profiling of the root zone (Yamaguchi and Sharp, 2010). Transcriptomic analysis was conducted on four regions of the root tip, designated as regions 1–4, defined by their differing spatial growth response to water deficit (Spollen et al., 2008). Only 7.5% of the differentially expressed transcripts were found to be the same between regions 1 and 2, indicating that the transcriptional response was dependent on the position along the root apex. A proteomic analysis of the cell wall proteins identified an increase in cell wall extension proteins and apoplastic reactive oxygen species in the most apical region of the root where root elongation rate is maintained (Zhu et al., 2007). This raises the possibility that these compounds regulate extension properties contributing to the maintenance of root elongation in response to water deficit. This work has led to important advances in understanding the regulation and adaptation of root growth to water deficit and highlights the importance of studying specific regions of the root. These studies clearly demonstrate the importance of combining physiological data with functional genomic data as well as emphasizing the importance of using spatial resolution.

Other studies integrating spatially resolved ‘omics in cereals are limited. Using laser-capture microdissection (LCM) spatially resolved transcriptomics was utilized to gain insight into the development of the maize leaf (Li et al., 2010).Along the developmental gradient, 64% of genes were differentially expressed, and 21% were differentially expressed between the bundle sheath and mesophyll cells. This study utilized the completed maize genome sequence and high-throughput Illumina sequencing for spatial profiling of the maize leave, providing novel information for understanding photosynthetic development in C4 plants. This information serves as a foundation for systems biology approaches and provides strong evidence for the importance of spatial profiling in plant studies.

## CONCLUSIONS AND FUTURE PERSPECTIVES

To increase our understanding of a plant’s response to abiotic stress and identify the mechanisms involved in abiotic stress tolerance it is important to study both the physiology and molecular networks that are involved. The use of functional genomic tools in abiotic stress research has substantially extended our knowledge about the basic biology and physiology of abiotic stress responses, however, it has not yet allowed the identification of reproducible markers that could be used to determine if, and to what extent, the plant was under stress. By utilizing advances in both phenomics and functional genomics technologies it is becoming possible to identify cell and tissue specific changes in the molecular and biochemical networks in response to abiotic stress. This will lead to an increased understanding of the molecular mechanisms responsible for abiotic stress tolerance and the generation of abiotic stress tolerant crops.

## Conflict of Interest Statement

The authors declare that the research was conducted in the absence of any commercial or financial relationships that could be construed as a potential conflict of interest.

## Footnotes

1 http://faostat.fao.org

2 http://www.fao.org/wsfs/world-summit/en/

## References

[B1] Amme S. Rutten T. Melzer M. Sonsmann G. Vissers J. P. C. Schlesier B. (2005). A proteome approach defines protective functions of tobacco leaf trichomes. *Proteomics* 5 2508–2518 15984042 10.1002/pmic.200401274

[B2] Barley Sequencing Consortium T. I. B. G. S. (2012). A physical, genetic and functional sequence assembly of the barley genome. *Nature* 491 711–716 23075845 10.1038/nature11543

[B3] Baxter I. (2009). Ionomics: studying the social network of mineral nutrients. *Curr. Opin. Plant Biol.* 12 381–386 19481970 10.1016/j.pbi.2009.05.002 PMC2701637

[B4] Becker J. S. (2010). ``Imaging of metals, metalloids, and non-metals by laser ablation inductively coupled plasma mass spectrometry (LA-ICP-MS) in biological tissues,'' in *Mass Spectrometry Imaging: Principles and Protocols*, eds Rubakhin S. S. Sweedler J. V. (Totowa: Humana Press Inc) 51–82 10.1007/978-1-60761-746-4_3 20680584

[B5] Beckles D. Roessner U. (2012). ``Plant metabolomics – applications and opportunities for agricultural biotechnology,'' in *Plant Biotechnology and Agriculture: Prospects for the 21st Century*, eds Altmann A. Hasegawa. P. M. (London: Elsevier) 67–78

[B6] Bernstein N. Kafkafi U. (2002). ``Root growth under salinity stress,'' in *Plant Roots: The Hidden Half III edition*, eds Waisel Y. Eshel A. Kafkafi U. (New York: Marcel Dekker, Inc.) 787–805

[B7] Borges F. Gardner R. Lopes T. Calarco J. P. Boavida L. C. Slotkin R. K. (2012). FACS-based purification of *Arabidopsis* microspores, sperm cells and vegetative nuclei. *Plant Methods* 8 44 10.1186/1746-4811-8-44 PMC3502443 23075219

[B8] Borisjuk L. Rolletschek H. Walenta S. Panitz R. Wobus U. Weber H. (2003). Energy status and its control on embryogenesis of legumes: ATP distribution within Vicia faba embryos is developmentally regulated and correlated with photosynthetic capacity. *Plant J.* 36 318–329 14617089 10.1046/j.1365-313x.2003.01879.x

[B9] Brady S. M. Orlando D. A. Lee J.-Y. Wang J. Y. Koch J. Dinneny J. R. (2007). A high-resolution root spatiotemporal map reveals dominant expression patterns. *Science* 318 801–806 17975066 10.1126/science.1146265

[B10] Cheng Y. Qi Y. Zhu Q. Chen X. Wang N. Zhao X. (2009). New changes in the plasma-membrane-associated proteome of rice roots under salt stress. *Proteomics* 9 3100–3114 19526560 10.1002/pmic.200800340

[B11] Cramer G. R. Ergul A. Grimplet J. Tillett R. L. Tattersall E. A. R. Bohlman M. C. (2007). Water and salinity stress in grapevines: early and late changes in transcript and metabolite profiles. *Funct. Integr. Genomics* 7 111–134 17136344 10.1007/s10142-006-0039-y

[B12] Cramer G. R. Urano K. Delrot S. Pezzotti M. Shinozaki K. (2011). Effects of abiotic stress on plants: a systems biology perspective. *BMC Plant Biol.* 11:163 10.1186/1471-2229-11-163 PMC3252258 22094046

[B13] Dassanayake M. Oh D. H. Haas J. S. Hernandez A. Hong H. Ali S. (2011). The genome of the extremophile crucifer *Thellungiella parvula*. *Nat. Genet.* 43 913–918 21822265 10.1038/ng.889 PMC3586812

[B14] Deyholos M. K. (2010). Making the most of drought and salinity transcriptomics. *Plant Cell Environ.* 33 648–654 20002333 10.1111/j.1365-3040.2009.02092.x

[B15] Diks S. H. Peppelenbosch M. P. (2004). Single cell proteomics for personalised medicine. *Trends Mol. Med.* 10 574–577 15567324 10.1016/j.molmed.2004.10.005

[B16] Dinneny J. R. (2010). Analysis of the salt-stress response at cell-type resolution. *Plant Cell Environ.* 33 543–551 19843253 10.1111/j.1365-3040.2009.02055.x

[B17] Dinneny J. R. Long T. A. Wang J. Y. Jung J. W. Mace D. Pointer S. (2008). Cell identity mediates the response of *Arabidopsis* roots to abiotic stress. *Science* 320 942–945 18436742 10.1126/science.1153795

[B18] Dong C. H. Li C. Yan X. H. Huang S. M. Huang J. Y. Wang L. J. (2012). Gene expression profiling of *Sinapis alba* leaves under drought stress and rewatering growth conditions with Illumina deep sequencing. *Mol. Biol. Rep.* 39 5851–5857 22207172 10.1007/s11033-011-1395-9

[B19] Dugas D. V. Monaco M. K. Olsen A. Klein R. R. Kumari S. Ware D. (2011). Functional annotation of the transcriptome of *Sorghum bicolor* in response to osmotic stress and abscisic acid. *BMC Genomics* 12:514 10.1186/1471-2164-12-514 PMC3219791 22008187

[B20] Evrard A. Bargmann B. O. Birnbaum K. D. Tester M. Baumann U. Johnson A. A. (2012). Fluorescence-activated cell sorting for analysis of cell type-specific responses to salinity stress in *Arabidopsis* and rice. *Methods Mol. Biol.* 913 265–276 22895766 10.1007/978-1-61779-986-0_18 PMC4164160

[B21] Fan X. D. Wang J. Q. Yang N. Dong Y. Y. Liu L. Wang F. W. (2013). Gene expression profiling of soybean leaves and roots under salt, saline-alkali and drought stress by high-throughput Illumina sequencing. *Gene* 512 392–402 23063936 10.1016/j.gene.2012.09.100

[B22] Garg B. Puranik S. Misra S. Nath Tripathi B. Prasad M. (2013). Transcript profiling identifies novel transcripts with unknown functions as primary response components to osmotic stress in wheat (*Triticum aestivum L*.). *Plant Cell Tiss. Organ Cult.* 113 91–101

[B23] Gewin V. (2010). Food: an underground revolution. *Nature* 466 552–553 20671689 10.1038/466552a

[B24] Gillies S. A. Futardo A. Henry R. J. (2012). Gene expression in the developing aleurone and starchy endosperm of wheat. *Plant Biotechnol. J.* 10 668–679 22672716 10.1111/j.1467-7652.2012.00705.x

[B25] Grassl J. Taylor N. L. Millar A. H. (2011). Matrix-assisted laser desorption/ionisation mass spectrometry imaging and its development for plant protein imaging. *Plant Methods* 7 21 10.1186/1746-4811-7-21 PMC3141805 21726462

[B26] Gruber V. Blanchet S. Diet A. Zahaf O. Boualem A. Kakar K. (2009). Identification of transcription factors involved in root apex responses to salt stress in *Medicago truncatula*. *Mol. Genet. Genomics* 281 55–66 18987888 10.1007/s00438-008-0392-8 PMC2757595

[B27] Guo G. F. Ge P. Ma C. Y. Li X. H. Lv D. W. Wang S. L. (2012). Comparative proteomic analysis of salt response proteins in seedling roots of two wheat varieties. *J. Proteomics* 75 1867–1885 22245046 10.1016/j.jprot.2011.12.032

[B28] Hare D. J. Lee J. K. Beavis A. D. Van Gramberg A. George J. Adlard P. A. (2012). Three-dimensional atlas of iron, copper, and zinc in the mouse cerebrum and brainstem. *Anal. Chem.* 84 3990–3997 22462591 10.1021/ac300374x

[B29] Hauser F. Horie T. (2010). A conserved primary salt tolerance mechanism mediated by HKT transporters: a mechanism for sodium exclusion and maintenance of high K^+^/Na^+^ ratio in leaves during salinity stress. *Plant Cell Environ.* 33 552–565 19895406 10.1111/j.1365-3040.2009.02056.x

[B30] Holmes-Davis R. Tanaka C. K. Vensel W. H. Hurkman W. J. Mccormick S. (2005). Proteome mapping of mature pollen of *Arabidopsis thaliana*. *Proteomics* 5 4864–4884 16247729 10.1002/pmic.200402011

[B31] Horie T. Hauser F. Schroeder J. I. (2009). HKT transporter-mediated salinity resistance mechanisms in *Arabidopsis* and monocot crop plants. *Trends Plant Sci.* 14 660–668 19783197 10.1016/j.tplants.2009.08.009 PMC2787891

[B32] Horie T. Sugawara M. Okada T. Taira K. Kaothien-Nakayama P. Katsuhara M. (2011). Rice sodium-insensitive potassium transporter, OsHAK5, confers increased salt tolerance in tobacco BY2 cells. *J. Biosci. Bioeng.* 111 346–356 21084222 10.1016/j.jbiosc.2010.10.014

[B33] Horn P. J. Korte A. R. Neogi P. B. Love E. Fuchs J. Strupat K. (2012). Spatial mapping of lipids at cellular resolution in embryos of cotton. *Plant Cell* 24 622–636 22337917 10.1105/tpc.111.094581 PMC3315237

[B34] Jain M. (2011). A next-generation approach to the characterization of a non-model plant transcriptome. *Curr. Sci.* 101 1435–1439

[B35] Jain M. (2012). Next-generation sequencing technologies for gene expression profiling in plants. *Brief. Funct. Genomics* 11 63–70 22155524 10.1093/bfgp/elr038

[B36] James R. A. Blake C. Zwart A. B. Hare R. A. Rathjen A. J. Munns R. (2012). Impact of ancestral wheat sodium exclusion genes Nax1 and Nax2 on grain yield of durum wheat on saline soils. *Funct. Plant Biol.* 39 609–618 10.1071/FP12121 32480813

[B37] James R. A. Davenport R. J. Munns R. (2006). Physiological characterization of two genes for Na^+^ exclusion in durum wheat, Nax1 and Nax2. *Plant Physiol.* 142 1537–1547 17028150 10.1104/pp.106.086538 PMC1676036

[B38] James R. A. Von Caemmerer S. Condon A. G. T. Zwart A. B. Munns R. (2008). Genetic variation in tolerance to the osmotic stress component of salinity stress in durum wheat. *Funct. Plant Biol.* 35 111–123 10.1071/FP07234 32688762

[B39] Jamil A. Riaz S. Ashraf M. Foolad M. R. (2011). Gene expression profiling of plants under salt stress. *Crit. Rev. Plant Sci.* 30 435–458

[B40] Jiang X. Leidi E. O. Pardo J. M. (2010). How do vacuolar NHX exchangers function in plant salt tolerance? *Plant Signal. Behav.* 5 792–795 20495345 10.4161/psb.5.7.11767 PMC3014531

[B41] Jun J. H. Song Z. H. Liu Z. J. Nikolau B. J. Yeung E. S. Lee Y. J. (2010). High-spatial and high-mass resolution imaging of surface metabolites of *Arabidopsis thaliana* by laser desorption-ionization mass spectrometry using colloidal silver. *Anal. Chem.* 82 3255–3265 20235569 10.1021/ac902990p

[B42] Kamal A. H. M. Cho K. Kim D. E. Uozumi N. Chung K. Y. Lee S. Y. (2012). Changes in physiology and protein abundance in salt-stressed wheat chloroplasts. *Mol. Biol. Rep.* 39 9059–9074 22736107 10.1007/s11033-012-1777-7

[B43] Kaspar S. Peukert M. Svatos A. Matros A. Mock H. P. (2011). MALDI-imaging mass spectrometry – an emerging technique in plant biology. *Proteomics* 11 1840–1850 21462348 10.1002/pmic.201000756

[B44] Kawaura K. Mochida K. Ogihara Y. (2006). Microarray analysis of salt-responsive genes in common wheat. *Genes Genet. Syst.* 81 437–437

[B45] Kawaura K. Mochida K. Ogihara Y. (2008). Genome-wide analysis for identification of salt-responsive genes in common wheat. *Funct. Integr. Genomics* 8 277–286 18320247 10.1007/s10142-008-0076-9

[B46] Keerberg O. Ivanova H. Keerberg H. Parnik T. Talts P. Gardestrom P. (2011). Quantitative analysis of photosynthetic carbon metabolism in protoplasts and intact leaves of barley. Determination of carbon fluxes and pool sizes of metabolites in different cellular compartments. *Biosystems* 103 291–301 21055441 10.1016/j.biosystems.2010.10.012

[B47] Kim D. Y. Hong M. J. Jang J. H. Seo Y. W. (2012). cDNA-AFLP analysis reveals differential gene expression in response to salt stress in Brachypodium distachyon. *Genes Genomics* 34 475–484

[B48] Kosova K. Vitamvas P. Prasil I. T. Renaut J. (2011). Plant proteome changes under abiotic stress – contribution of proteomics studies to understanding plant stress response. *J. Proteomics* 74 1301–1322 21329772 10.1016/j.jprot.2011.02.006

[B49] Kreps J. A. Wu Y. Chang H.-S. Zhu T. Wang X. Harper J. F. (2002). Transcriptome changes for *Arabidopsis* in response to salt, osmotic, and cold stress. *Plant Physiol.* 130 2129–2141 12481097 10.1104/pp.008532 PMC166725

[B50] Kryvych S. Kleessen S. Ebert B. Kersten B. Fisahn J. (2011). Proteomics – the key to understanding systems biology of *Arabidopsis* trichomes. *Phytochemistry* 72 1061–1070 20952039 10.1016/j.phytochem.2010.09.003

[B51] Langridge P. Fleury D. (2011). Making the most of `omics' for crop breeding. *Trends Biotechnol.* 29 33–40 21030098 10.1016/j.tibtech.2010.09.006

[B52] Lee Y. J. Perdian D. C. Song Z. H. Yeung E. S. Nikolau B. J. (2012). Use of mass spectrometry for imaging metabolites in plants. *Plant J.* 70 81–95 22449044 10.1111/j.1365-313X.2012.04899.x

[B53] Li P. Ponnala L. Gandotra N. Wang L. Si Y. Tausta S. L. (2010). The developmental dynamics of the maize leaf transcriptome. *Nat. Genet.* 42 1060–1067 21037569 10.1038/ng.703

[B54] Lindsay M. P. Lagudah E. S. Hare R. A. Munns R. (2004). A locus for sodium exclusion (Nax1), a trait for salt tolerance, mapped in durum wheat. *Funct. Plant Biol.* 31 1105–1114 10.1071/FP04111 32688978

[B55] Lister R. O’Malley R. C. Tonti-Filippini J. Gregory B. D. Berry C. C. Millar A. H. (2008). Highly integrated single-base resolution maps of the epigenome in *Arabidopsis*. *Cell* 133 523–536 18423832 10.1016/j.cell.2008.03.029 PMC2723732

[B56] Long T. A. (2011). Many needles in a haystack: cell-type specific abiotic stress responses. *Curr. Opin. Plant Biol.* 14 325–331 21550295 10.1016/j.pbi.2011.04.005

[B57] Mian A. Oomen R. Isayenkov S. Sentenac H. Maathuis F. J. M. Very A. A. (2011). Over-expression of an Na(+)- and K(+)-permeable HKT transporter in barley improves salt tolerance. *Plant J.* 68 468–479 21749504 10.1111/j.1365-313X.2011.04701.x

[B58] Molina C. Rotter B. Horres R. Udupa S. M. Besser B. Bellarmino L. (2008). SuperSAGE: the drought stress-responsive transcriptome of chickpea roots. *BMC Genomics* 9:553 10.1186/1471-2164-9-553 PMC2628679 19025623

[B59] Mortazavi A. Williams B. A. Mccue K. Schaeffer L. Wold B. (2008). Mapping and quantifying mammalian transcriptomes by RNA-Seq. *Nat. Methods* 5 621–628 18516045 10.1038/nmeth.1226 PMC13303166

[B60] Mott I. W Wang R. R. C. (2007). Comparative transcriptome analysis of salt-tolerant wheat germplasm lines using wheat genome arrays. *Plant Sci.* 173 327–339

[B61] Munns R. (2002). Comparative physiology of salt and water stress. *Plant Cell Environ.* 25 239–250 11841667 10.1046/j.0016-8025.2001.00808.x

[B62] Munns R. Hare R. A. James R. A. Rebetzke G. J. (2000). Genetic variation for improving the salt tolerance of durum wheat. *Austr. J. Agric. Res.* 51 69–74

[B63] Munns R. Schachtman D. P. Condon A. G. (1995). The significance of a 2-phase growth-response to salinity in wheat and barley. *Aust. J. Plant Physiol.* 22 561–569

[B64] Munns R. Tester M. (2008). Mechanisms of salinity tolerance. *Annu. Rev. Plant Biol.* 59 651–681 18444910 10.1146/annurev.arplant.59.032607.092911

[B65] Nagalakshmi U. Wang Z. Waern K. Shou C. Raha D. Gerstein M. (2008). The transcriptional landscape of the yeast genome defined by RNA sequencing. *Science* 320 1344 –1349 18451266 10.1126/science.1158441 PMC2951732

[B66] Obata T. Fernie A. R. (2012). The use of metabolomics to dissect plant responses to abiotic stresses. *Cell. Mol. Life Sci.* 69 3225–3243 22885821 10.1007/s00018-012-1091-5 PMC3437017

[B67] Paterson A. H. Bowers J. E. Bruggmann R. Dubchak I. Grimwood J. Gundlach H. (2009). The Sorghum bicolor genome and the diversification of grasses. *Nature* 457 551–556 19189423 10.1038/nature07723

[B68] Petersson S. V. Johansson A. I. Kowalczyk M. Makoveychuk A. Wang J. Y. Moritz T. (2009). An auxin gradient and maximum in the *Arabidopsis* root apex shown by high-resolution cell-specific analysis of IAA distribution and synthesis. *Plant Cell* 21 1659–1668 19491238 10.1105/tpc.109.066480 PMC2714926

[B69] Peukert M. Matros A. Lattanzio G. Kaspar S. Abadia J. Mock H. P. (2012). Spatially resolved analysis of small molecules by matrix-assisted laser desorption/ionization mass spectrometric imaging (MALDI-MSI). *New Phytol.* 193 806–815 22126099 10.1111/j.1469-8137.2011.03970.x

[B70] Plett D. C. Moller I. S. (2010). Na plus transport in glycophytic plants: what we know and would like to know. *Plant Cell Environ.* 33 612–626 19968828 10.1111/j.1365-3040.2009.02086.x

[B71] Project I. R. G. S. (2005). The map-based sequence of the rice genome. *Nature* 436 793–800 16100779 10.1038/nature03895

[B72] Pu L. Brady S. (2010). Systems biology update: cell type-specific transcriptional regulatory networks. *Plant Physiol.* 152 411–419 19965967 10.1104/pp.109.148668 PMC2815853

[B73] Rahnama A. Munns R. Poustini K. Watt M. (2011). A screening method to identify genetic variation in root growth response to a salinity gradient. *J. Exp. Bot.* 62 69–77 21118825 10.1093/jxb/erq359

[B74] Rajendran K. Tester M. Roy S. J. (2009). Quantifying the three main components of salinity tolerance in cereals. *Plant Cell Environ.* 32 237–249 19054352 10.1111/j.1365-3040.2008.01916.x

[B75] Richards R. A. Rebetzke G. J. Watt M. Condon A. G. Spielmeyer W. Dolferus R. (2010). Breeding for improved water productivity in temperate cereals: phenotyping, quantitative trait loci, markers and the selection environment. *Funct. Plant Biol.* 37 85–97

[B76] Roessner U. Beckles D. M. (2009). ``Metabolite measurements,'' in *Plant Metabolic Networks*, ed. Schwender J. (New York: Springer) 39

[B77] Rogers E. D. Jackson T. Moussaieff A. Aharoni A. Benfey P. N. (2012). Cell type-specific transcriptional profiling: implications for metabolite profiling. *Plant J.* 70 5–17 22449039 10.1111/j.1365-313X.2012.04888.x PMC3315153

[B78] Roy A. Rushton P. J. Rohila J. S. (2011a). The potential of proteomics technologies for crop improvement under drought conditions. *Crit. Rev. Plant Sci.* 30 471–490

[B79] Roy S. J. Tucker E. J. Tester M. (2011b). Genetic analysis of abiotic stress tolerance in crops. *Curr. Opin. Plant Biol.* 14 232–239 21478049 10.1016/j.pbi.2011.03.002

[B80] Sarhadi E. Bazargani M. M. Sajise A. G. Abdolahi S. Vispo N. A. Arceta M. (2012). Proteomic analysis of rice anthers under salt stress. *Plant Physiol. Biochem.* 58 280–287 22868211 10.1016/j.plaphy.2012.07.013

[B81] Schad M. Mungur R. Fiehn O. Kehr J. (2005). Metabolic profiling of laser microdissected vascular bundles of *Arabidopsis thaliana*. *Plant Methods* 1 2 10.1186/1746-4811-1-2 PMC1266046 16270917

[B82] Schirle M. Heurtier M. A. Kuster B. (2003). Profiling core proteomes of human cell lines by one-dimensional PAGE and liquid chromatography-tandem mass spectrometry. *Mol. Cell. Proteomics* 2 1297–1305 14532353 10.1074/mcp.M300087-MCP200

[B83] Schnable P. S. Ware D. Fulton R. S. Stein J. C. Wei F. Pasternak S. (2009). The B73 maize genome: complexity, diversity, and dynamics. *Science* 326 1112–1115 19965430 10.1126/science.1178534

[B84] Sharp R. E. Silk W. K. Hsiao T. C. (1988). Growth of the maize primary root at low water potentials.1 . Spatial-distribution of expansive growth. *Plant Physiol.* 87 50–57 16666126 10.1104/pp.87.1.50 PMC1054698

[B85] Shavrukov Y. Gupta N. K. Miyazaki J. Baho M. N. Chalmers K. J. Tester M. (2010). HvNax3-a locus controlling shoot sodium exclusion derived from wild barley (*Hordeum vulgare* ssp spontaneum). *Funct. Integr. Genomics* 10 277–291 20076983 10.1007/s10142-009-0153-8

[B86] Shelden M. C. Roessner U. Sharp R. E. Tester M. Bacic A. (2013). Genetic variation in the root growth response of barley genotypes to salinity stress. *Funct. Plant Biol.* 10.1071/FP12290 [Epub ahead of print] 32481128

[B87] Spollen W. G. Tao W. Valliyodan B. Chen K. Hejlek L. G. Kim J. J. (2008). Spatial distribution of transcript changes in the maize primary root elongation zone at low water potential. *BMC Plant Biol.* 8:32 10.1186/1471-2229-8-32 PMC2364623 18387193

[B88] Studer B. Byrne S. Nielsen R. O. Panitz F. Bendixen C. Islam M. S. (2012). A transcriptome map of perennial ryegrass (*Lolium perenne* L.). *BMC Genomics* 13:140 10.1186/1471-2164-13-140 PMC3483695 22513206

[B89] Tamura K. Yonemaru J. (2010). Next-generation sequencing for comparative transcriptomics of perennial ryegrass (*Lolium perenne* L.) and meadow fescue (*Festuca pratensis* Huds.) during cold acclimation. *Grassland Sci.* 56 230–239

[B90] Tester M. Langridge P. (2010). Breeding technologies to increase crop production in a changing world. *Science* 327 818–822 20150489 10.1126/science.1183700

[B91] Ueda A. Kathiresan A. Bennett J. Takabe T. (2006). Comparative transcriptome analyses of barley and rice under salt stress. *Theor. Appl. Genet.* 112 1286–1294 16496119 10.1007/s00122-006-0231-4

[B92] Ueda A. Kathiresan A. Inada M. Narita Y. Nakamura T. Shi W. M. (2004). Osmotic stress in barley regulates expression of a different set of genes than salt stress does. *J. Exp. Bot.* 55 2213–2218 15361537 10.1093/jxb/erh242

[B93] Urano K. Kurihara Y. Seki M. Shinozaki K. (2010). `Omics' analyses of regulatory networks in plant abiotic stress responses. *Curr. Opin. Plant Biol.* 13 132–138 20080055 10.1016/j.pbi.2009.12.006

[B94] Vidal R. O. Do Nascimento L. C. Mondego J. M. C. Pereira G. A. G. Carazzolle M. F. (2012). Identification of SNPs in RNA-seq data of two cultivars of Glycine max (soybean) differing in drought resistance. *Genet. Mol. Biol.* 35 331–334 22802718 10.1590/S1415-47572012000200014 PMC3392885

[B95] Vogel J. P. Garvin D. F. Mockler T. C. Schmutz J. Rokhsar D. Bevan M. W. (2010). Genome sequencing and analysis of the model grass *Brachypodium distachyon*. *Nature* 463 763–768 20148030 10.1038/nature08747

[B96] Vrkoslav V. Muck A. Cvacka J. Svatos A. (2010). MALDI imaging of neutral cuticular lipids in insects and plants. *J. Am. Soc. Mass Spectrom.* 21 220–231 19910210 10.1016/j.jasms.2009.10.003

[B97] Walia H. Wilson C. Condamine P. Ismail A. M. Xu J. Cui X. P. (2007). Array-based genotyping and expression analysis of barley cv. Maythorpe and Golden Promise. *BMC Genomics* 8:87 10.1186/1471-2164-8-87 PMC1851953 17394671

[B98] Walia H. Wilson C. Wada H. Condamine P. Cui X. P. Close T. J. (2006). Expression analysis of barley (*Hordeum vulgare* L.)**during salinity stress. *Funct. Integr. Genomics* 6 143–156 16450154 10.1007/s10142-005-0013-0

[B99] Wan J. R. Torres M. Ganapathy A. Thelen J. Dague B. B. Mooney B. (2005). Proteomic analysis of soybean root hairs after infection by *Bradyrhizobium japonicum*. *Mol. Plant Microbe Interact.* 18 458–467 15915644 10.1094/MPMI-18-0458

[B100] Wang Y. Zeng X. Iyer N. J. Bryant D. W. Mockler T. C. Mahalingam R. (2012). Exploring the switchgrass transcriptome using second-generation sequencing technology. *PLoS ONE* 7:e34225 10.1371/journal.pone.0034225 PMC3315583 22479570

[B101] Weber A. P. Weber K. L. Carr K. Wilkerson C. Ohlrogge J. B. (2007). Sampling the *Arabidopsis* transcriptome with massively parallel pyrosequencing. *Plant Physiol.* 144 32 –42 17351049 10.1104/pp.107.096677 PMC1913805

[B102] Widodo Patterson J. H. Newbigin E. Tester M. Bacic A. Roessner U. (2009). Metabolic responses to salt stress of barley (*Hordeum vulgare* L.)**cultivars, Sahara and Clipper, which differ in salinity tolerance. *J. Exp. Bot.* 60 4089–4103 19666960 10.1093/jxb/erp243 PMC2755029

[B103] Wienkoop S. Zoeller D. Ebert B. Simon-Rosin U. Fisahn J. Glinski M. (2004). Cell-specific protein profiling in *Arabidopsis thaliana* trichomes: identification of trichome-located proteins involved in sulfur metabolism and detoxification. *Phytochemistry* 65 1641–1649 15276459 10.1016/j.phytochem.2004.03.026

[B104] Xue S. W. Yao X. Luo W. Jha D. Tester M. Horie T. (2011). AtHKT1;1 mediates nernstian sodium channel transport properties in *Arabidopsis* root stelar cells. *PLoS ONE* 6:e24725 10.1371/journal.pone.0024725 PMC3170383 21931830

[B105] Yamaguchi M. Sharp R. E. (2010). Complexity and coordination of root growth at low water potentials: recent advances from transcriptomic and proteomic analyses. *Plant Cell Environ.* 33 590–603 19895398 10.1111/j.1365-3040.2009.02064.x

[B106] Yi L. Liang Z. T. Peng Y. Yao X. Chen H. B. Zhao Z. Z. (2012). Tissue-specific metabolite profiling of alkaloids in *Sinomenii Caulis* using laser microdissection and liquid chromatography-quadrupole/time of flight-mass spectrometry. *J. Chromatogr. A* 1248 93–103 22721764 10.1016/j.chroma.2012.05.058

[B107] Zhang H. Han B. Wang T. Chen S. X. Li H. Y. Zhang Y. H. (2012). Mechanisms of plant salt response: insights from proteomics. *J. Proteome Res.* 11 49–67 22017755 10.1021/pr200861w

[B108] Zhao Z. X. Zhang W. Stanley B. A. Assmann S. M. (2008). Functional proteomics of *Arabidopsis thaliana* guard cells uncovers new stomatal signaling pathways. *Plant Cell* 20 3210–3226 19114538 10.1105/tpc.108.063263 PMC2630442

[B109] Zhu J. Ingram P. A. Benfey P. N. Elich T. (2011). From lab to field, new approaches to phenotyping root system architecture. *Curr. Opin. Plant Biol.* 14 310–317 21530367 10.1016/j.pbi.2011.03.020

[B110] Zhu J. M. Alvarez S. Marsh E. L. Lenoble M. E. Cho I. J. Sivaguru M. (2007). Cell wall Proteome in the maize primary root elongation zone. II. Region-specific changes in water soluble and lightly ionically bound proteins under water deficit. *Plant Physiol.* 145 1533–1548 10.1104/pp.107.107250 PMC2151692 17951457

